# Variability of Serum Proteins in Chinese and Dutch Human Milk during Lactation

**DOI:** 10.3390/nu11030499

**Published:** 2019-02-27

**Authors:** Mohèb Elwakiel, Sjef Boeren, Jos A. Hageman, Ignatius M. Szeto, Henk A. Schols, Kasper A. Hettinga

**Affiliations:** 1 Food Quality and Design Group, Wageningen University & Research, Bornse Weilanden 9, 6708 WG Wageningen, The Netherlands; moheb.elwakiel@wur.nl; 2 Laboratory of Food Chemistry, Wageningen University & Research, Bornse Weilanden 9, 6708 WG Wageningen, The Netherlands; henk.schols@wur.nl; 3 Laboratory of Biochemistry, Wageningen University & Research, Stippeneng 4, 6708 WE Wageningen, The Netherlands; sjef.boeren@wur.nl; 4 Biometris-Applied Statistics, Wageningen University & Research, Droevendaalsesteeg 1, 6708 PB Wageningen, The Netherlands; jos.hageman@wur.nl; 5 Inner Mongolia Yili Industrial Group Co., Ltd., Jinshan Road 8, Hohhot 010110, China; szeto@yili.com

**Keywords:** mammary gland, immune-active proteins, proteases, protease inhibitors, digestive tract

## Abstract

To better understand the variability of the type and level of serum proteins in human milk, the milk serum proteome of Chinese mothers during lactation was investigated using proteomic techniques and compared to the milk serum proteome of Dutch mothers. This showed that total milk serum protein concentrations in Chinese human milk decreased over a 20-week lactation period, although with variation between mothers in the rate of decrease. Variation was also found in the composition of serum proteins in both colostrum and mature milk, although immune-active proteins, enzymes, and transport proteins were the most abundant for all mothers. These three protein groups account for many of the 15 most abundant proteins, with these 15 proteins covering more than 95% of the total protein concentrations, in both the Chinese and Dutch milk serum proteome. The Dutch and Chinese milk serum proteome were also compared based on 166 common milk serum proteins, which showed that 22% of the 166 serum proteins differed in level. These differences were observed mainly in colostrum and concern several highly abundant proteins. This study also showed that protease inhibitors, which are highly correlated to immune-active proteins, are present in variable amounts in human milk and could be relevant during digestion.

## 1. Introduction

Human milk is the best source of nutrition for babies, enhances children’s immune system and influences the microbiota [1,2,3]. Health benefits have been linked to the presence and concentration of human milk components like oligosaccharides and proteins [4,5]. There are two distinct groups of proteins in human milk; caseins and milk serum proteins [6]. Human milk in early lactation consists of approximately 30% caseins and 70% serum proteins, with a 50:50 ratio typically found after a six month lactation period [6]. 

Serum proteins in human milk have been categorized according to their main and highly diverse biological functions [7,8]. It was found that immune-related proteins, transport proteins, and enzymes were present in the largest quantities, and their concentrations generally decrease over lactation [7,8]. Immune-active proteins not only protect infants against pathogenic microorganisms, but also confer passive immunity to the neonate until its own immune system has been fully developed [9,10,11]. Serum proteins in human milk also include an array of blood coagulation proteins, membrane proteins, signaling proteins, and protease inhibitors [9,10,11]. Protease inhibitors play a key role in the blood coagulation cascade and complement pathway [12,13,14], and might protect proteins against degradation by proteases in the mammary gland and even in the infant’s gastrointestinal tract [12,13,14,15,16,17,18]. 

There is a wide range of proteins (e.g., α_S1_-, β-, and κ-casein, lactoferrin, immunoglobulins, serum albumin, and α-lactalbumin) in relatively high concentrations in human milk [19]. Most milk proteins are synthesized in the mammary gland, except for immunoglobulins and serum albumin [19]. Serum albumin can enter milk via the paracellular pathway and immunoglobulins are transported from blood through mammary epithelial cells by a receptor-mediated mechanism [19]. Caseins are transport proteins that form micelles, and these micelles are capable of binding—and thereby transporting—minerals. Caseins can easily be digested in the infant’s gastrointestinal tract [15,16,17,18], being a valuable source of amino acids and minerals, which can easily be absorbed. Milk serum proteins such as lactoferrin, immunoglobulins, serum albumin, and α-lactalbumin cover 90% of the milk serum proteome in abundance [20]. The milk serum protein α-lactalbumin is required for the synthesis of lactose, supplies infants with large amounts of tryptophan, and facilitates the absorption of essential minerals [21]. Several other milk serum proteins, like lactoferrin and immunoglobulins, protect infants against pathogens and decrease the risk of having acute or chronic diseases [21,22]. Lactoferrin, a globular glycoprotein of the transferrin family, ends up in the infant’s feces, and was shown to influence the microbiota composition of neonates [22]. Human milk is also a rich source of antibodies or immunoglobulins, which are able to recognize and bind to unique epitopes of pathogens, preventing their colonization [23,24,25]. Serum albumin is a protein mainly involved in the transportation of hormones, fatty acids, and other milk components [21]. 

Individual differences in milk serum proteins between mothers have been reported, where it was found that there was a large overlap in identified proteins in human milk among mothers, whereas there were also major quantitative changes, both between mothers and over time [7]. Given the various potential benefits of milk serum proteins, it would be of interest to obtain insights in the variability of serum proteins in human milk from mothers from other geographical and ethnic origin. 

Therefore, the main objective of this study was to investigate the milk serum proteome of seven Chinese mothers and to investigate the variability in type and level of serum proteins in Chinese human milk over a 20-week lactation period using liquid chromatography and mass spectrometry (LC-MS/MS). Additionally, the type and level of serum proteins in Chinese human milk were compared to those in colostrum and mature milk from Dutch mothers.

## 2. Materials and Methods

### 2.1. Study Setup and Sample Collection

Chinese participants were recruited in the Hohhot region, China, between August 2014 and November 2015 by the Yili Innovation Center (Hohhot, China). Yili organized the collection of the human milk, including sampling using a human milk pump. For every time point, a volume of 10 mL was collected in a polypropylene bottles. Milk bottles were shaken gently, aliquoted directly into 2 mL Eppendorf tubes, and stored at −20 °C. Milk samples from seven healthy mothers who delivered term (38–42 weeks) infants were assessed in weeks 1, 2, 4, 8, 12, and 20 postpartum. Human milk collection was approved by the Chinese Ethics Committee of Registering Clinical Trials (ChiECRCT-20150017). Written informed consent was obtained from all mothers. Milk collection and analysis of the milk of four Dutch mothers over a 24-week lactation period was described preciously and was a collaboration with the Dutch Human Milk Bank (Amsterdam, The Netherlands) [7]. Healthy women who delivered singleton term infants (38–42 weeks) were eligible for that study. The data from these analyses were re-used and made compatible with the Chinese data within this research to facilitate direct comparison, as explained further in Section 2.4 (Data Analysis).

### 2.2. Milk Serum Preparation and Concentrations

Human milk samples (5 mL) were fractionated, as described previously [10]. Briefly, the milk fat was removed by centrifugation (10 min, 1500 *g*, 4 °C) and the obtained skim milk was transferred to ultracentrifuge tubes. After ultracentrifugation (90 min, 100,000 *g*, 4 °C), the top layer represented the remaining milk fat still present, the middle layer was milk serum (with some free soluble caseins), and the bottom layer consisted of micellar casein. The free soluble caseins are part of the milk serum proteome. A comparative study previously showed that ultracentrifugation is the most effective method to separate caseins from serum proteins [26], although it is not possible to rule out low amounts of serum proteins in the casein pellet [6]. Milk serum concentrations were measured in duplicate using the bicinchoninic acid (BCA) protein assay kit (Thermo Scientific Pierce, Massachusetts, U.S.), to ensure that the same amount of protein (10 μg) was used for further sample preparation. Bovine serum albumin was used as standard for making a BCA calibration curve. 

### 2.3. Sample Preparation, Dimethyl Labeling, Protein Digestion, and Peptide Analysis

Milk serum samples were prepared for protein analysis using filter-aided sample preparation and dimethyl labeling, as described previously [27]. Milk serum (20 μL) was mixed with a buffer containing sodium dodecyl sulfate (SDS) for protein denaturation and dithiothreitol (DTT) to reduce the disulfide bridges in proteins, after which the samples were loaded on a Pall 3 K omega filter (10–20 kDa cutoff, OD003C34, Pall, Washington, U.S.) for protein digestion. The lysis buffer contained 0.1 M Tris/HCl pH 8.0 + 4% SDS + 0.1 M DTT to get a 1 μg/μL protein solution. Next, 180 μL of 0.05 M iodoacetamide/urea (0.1 M Tris/HCl pH 8 + 8 M urea) was used for protein alkylation. Samples were washed three times with 100 μL of 8 M urea, using centrifugation, followed by 110 μL of 50 mM ammonium bicarbonate (ABC). Then 0.5 μg trypsin in 100 μL ABC was added, followed by overnight incubation at room temperature while mildly shaking, and centrifuged to separate peptides from undigested material. The trypsin digested samples were then labeled, using distinct combinations of isotopic isomers of formaldehyde and cyanoborohydride, leading to a unique stable isotope composition of labeled peptide doublets with different masses [27]. After dimethyl labeling, the prepared samples were analyzed using LC-MS/MS, as described before [7]. For LC-MS/MS, a Prontosil 300-3-C18Hmagic C18AQ 200 Å analytical column was used, and the full scan FTMS spectra were measured in positive mode between *m*/*z* 380 and 1400 on a Thermo LTQ-Orbitrap XL. CID fragmented MS/MS scans of the four most abundant doubly- and triply-charged peaks in the FTMS scan were recorded in data-dependent mode in the linear trap (MS/MS threshold = 5.000). 

### 2.4. Data Analysis

The MS/MS spectra obtained were processed by the software package Maxquant 1.3.0.5 with the Andromeda search engine, as described previously [28]. Protein identification and quantification was done according to the literature [7]. Maxquant created a decoy database consisting of reversed sequences to calculate the false discovery rate (FDR). The FDR was set to 0.01 at the peptide and protein levels. The minimum required peptide length was six amino acids, and proteins were identified based on a minimum of two distinct peptides. The intensity–based absolute quantification (iBAQ) values were selected, representing the total peak intensity as determined by Maxquant for each protein and their values were corrected for the number of measurable peptides [7]. The iBAQ values have been reported to have a good correlation with known absolute protein amounts over at least four orders of magnitude [29]. For data normalization, iBAQ values for each protein were transformed into BCA equivalent milk serum protein concentrations, by dividing the iBAQ values of each protein in a sample by the summed iBAQ values of all protein within a sample, there were then multiplied with the corresponding milk serum protein concentration based on the BCA assay. To facilitate direct comparison between Chinese and Dutch data within this research, BCA equivalent values at time points 12 and 20 weeks postpartum were compared to weeks 16 and 24, respectively. Biological functions were assigned to all the serum proteins using the online UniprotKB database, as done previously [7]. To assign a specific function to multifunctional proteins, DAVID Bioinformatics Resource 6.7 was used additionally for further protein biological function classification and clarification [30].

### 2.5. Statistical Analysis

Statistical analysis was performed based upon previously described methods [7], with modifications. For the BCA equivalent values of each protein in Chinese and Dutch human milk over lactation, a regression line was fitted using R (Lucent Technologies, New York, NY, U.S.A.), summarizing the profile over time for each protein into an intercept and slope. The calculated intercepts are the protein BCA equivalent values at week 1, while the calculated slopes indicate the decrease or increase in BCA equivalent values per week. To determine the significant different milk serum proteins over the course of lactation per country, a comparison was made based on the calculated slope. Only BCA equivalent values of the common serum proteins found in both Chinese and Dutch human milk were used for comparison. The common serum proteins in Chinese and Dutch human milk were then evaluated based on the calculated intercept and slope using a two-tailed *t*-test, with a significance level set at α = 0.05. Next, these common milk serum proteins were compared in Chinese and Dutch human milk using a two-tailed *t*-test in Perseus [31], separately for each lactation week, with correction for multiple testing based on permutation-based FDR. The BCA equivalent values of serum proteins in Chinese and Dutch human milk were also summed per function and were then compared using a two-tailed *t*-test. To quantify the relation between biological function groups, Pearson correlation coefficients were calculated for summed BCA equivalent values and visualized in correlation matrix plots. Pearson correlation coefficients of >0.5 were considered good. All the serum proteins in Chinese and Dutch human milk were plotted in a graph in order to visualize the differences in serum proteins over the course of lactation.

## 3. Results

The objective of this study was to investigate the variability in the type and level of serum proteins in Chinese human milk over a 20-week lactation period. For this, the milk serum proteome of seven mothers over the course of lactation was investigated using LC-MS/MS. 

### 3.1. Level and Type of Milk Serum Proteins in Chinese Human Milk

**Figure 1:**
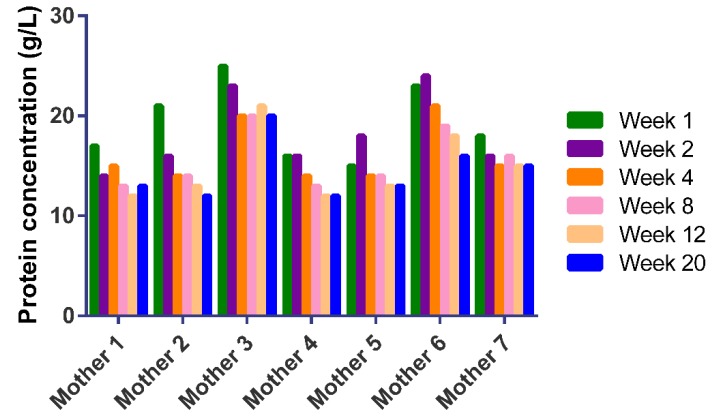
Total bicinchoninic acid (BCA) serum protein concentrations (g/L) in Chinese human milk per mother over a 20-week lactation period.

The total milk serum protein concentrations in Chinese human milk of the seven mothers over the course of lactation are presented in Figure 1. Concentrations ranging from 12 to 25 g/L decreased significantly (α < 0.05) over a 20-week lactation period, although with large individual variations (Figure 1). 

**Figure 2:**
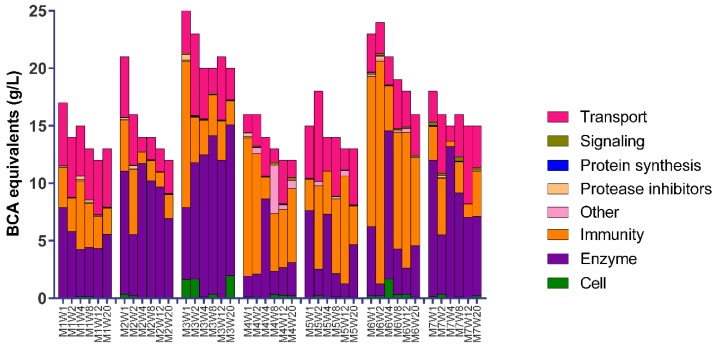
Serum protein composition in human milk of seven Chinese mothers over a 20-week lactation period, based on BCA equivalent values (g/L). The number after the M indicates the mother, and the numbers after the W (1 to 20) indicates the number of weeks postpartum.

Serum proteins in human milk were grouped based on their main biological functions (Supplementary Supporting information, data file). Not only the total protein concentrations, but also the protein composition differed among mothers and over lactation as measured after protein digestion and subsequent LC-MS/MS analysis (Figure 2). The figure shows that immune-active proteins, transport proteins, and enzymes were the most abundant for all mothers (Figure 2). The percentage of total protein attributable to these main biological functions, however, varied widely among mothers (Figure 2). Although the BCA equivalent values were always higher in colostrum than in mature milk, the rate of decline for the three main groups varied among mothers (Figure 2). 

**Figure 3:**
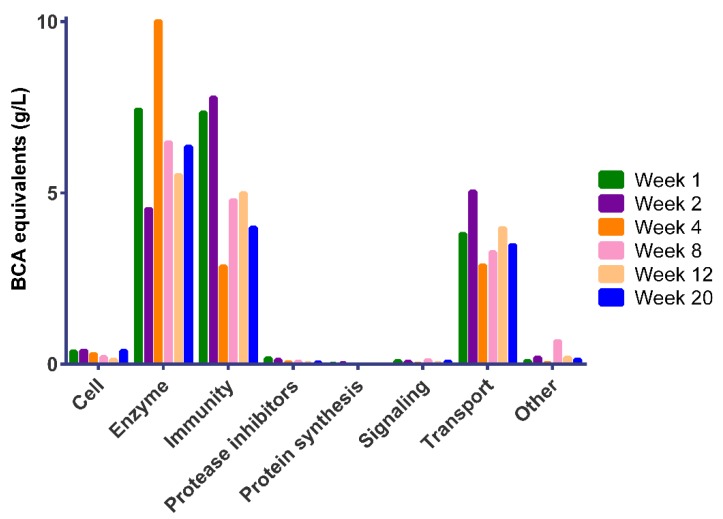
Averaged BCA equivalent values (g/L) of serum proteins for human milk from seven Chinese mothers categorized per biological function over a 20-week lactation period.

To facilitate the comparison between Chinese and Dutch human milk, data were averaged among mothers, as shown in Figure 3. The average total BCA equivalent values in Chinese human milk for enzymes, immune-active proteins, and transport proteins ranged over 4.5–10.0 g/L, 2.9–7.8 g/L, and 2.9–5.0 g/L, respectively (Figure 3). 

### 3.2. Comparison of the Chinese and Dutch Milk Serum Proteomes

The type and level of serum proteins in Chinese human milk were also compared to those in Dutch human milk. The raw data on Dutch human milk were reprocessed to be compatible with the Chinese data. The total BCA milk serum protein concentrations in Dutch human milk per mother and over the course of lactation are available as supplementary information (Figure S1). The total BCA equivalent values in Dutch human milk decreased over a 24-week lactation period from 21.6 to 13.6 g/L (Figure S2). Enzymes, immune-active proteins, and transport proteins were also the most abundant in Dutch human milk over the course of lactation (Figure S2). The BCA equivalent values for the groups enzymes, immune-active proteins, and transport proteins in Dutch human milk ranged over 4.5–9.0 g/L, 3.8–5.6 g/L, and 4.8–6.8 g/L, respectively. Although different patterns in Chinese and Dutch human milk can be observed, the difference was not significant between the same group of biological functions (data not shown), except for cell and signaling, where levels were higher in Chinese human milk. 

**Figure 4:**
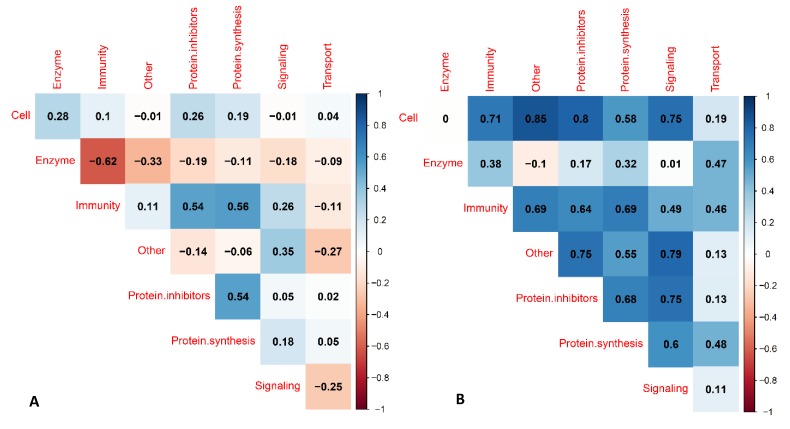
Calculated Pearson correlation coefficients between the different functional groups of serum proteins in Chinese and Dutch human milk, using the summed BCA equivalent values (g/L) over lactation. (**A**) Chinese human milk and (**B**) Dutch human milk.

The relations between the levels of different biological function groups of serum proteins within the Chinese and within the Dutch human milk populations were visualized in a correlation matrix plot (Figure 4).

### 3.3. Individual Milk Serum Proteins 

Totals of 469 and 200 serum proteins were measured in Chinese and Dutch human milk, respectively. The milk serum proteomes of different Chinese and Dutch mothers were compared based on 166 common milk serum proteins. The overall 15 most abundant milk serum proteins can be found in Table 1. 

In Dutch human milk, α_1_-antichymotrypsin belongs to the top 15 serum proteins instead of the transport protein fatty acid-binding protein (Table 1). Within the group enzymes, the highly abundant α-lactalbumin and bile salt-activated lipase are mainly responsible for the changes in this group in human milk over the course of lactation (Table 1). Many immune-active proteins, like lactoferrin, osteopontin, different types of immunoglobulins, polymeric immunoglobulin receptor, and clusterin, belong to the most abundant serum proteins in human milk (Table 1). The changes within the group of transport proteins over the course of lactation can mainly be explained by the caseins (Table 1). The caseins in Table 1 probably refer to the free, non-micellar casein, as the micellar casein should have been removed during the sample preparation (Table 1). With the majority of the caseins in milk being part of the micellar fraction, the caseins in Table 1 therefore do not reflect the levels of total casein.

**Figure 5:**
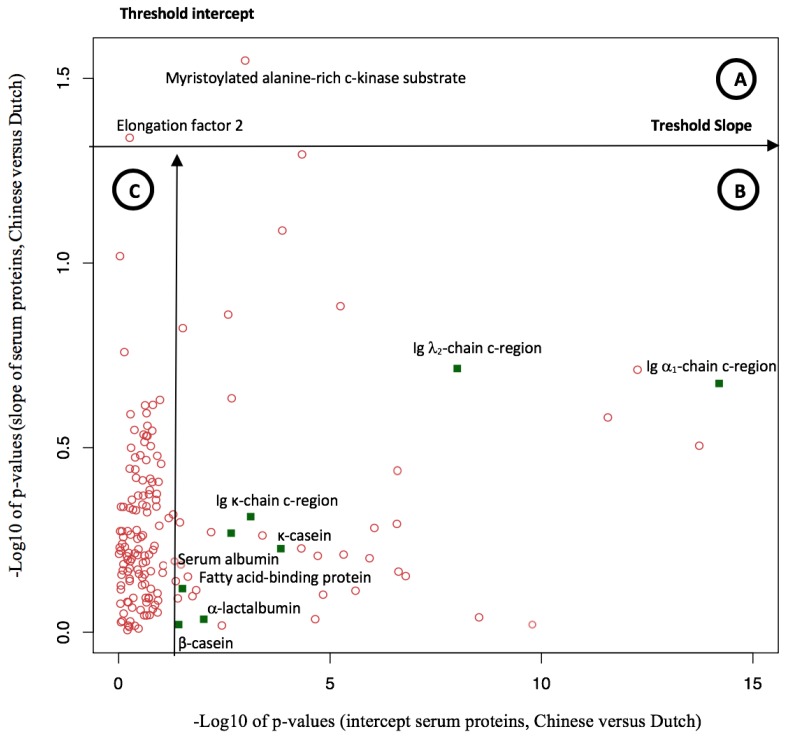
Comparison of the common serum proteins in Chinese and Dutch human milk during lactation. Green squares indicate the proteins displayed in Table 1. For each serum protein in Chinese and Dutch human milk over the course of lactation, a regression line was fitted, summarizing the profile for each protein into an intercept (representing week 1) and slope (representing rate of change over lactation). These profiles were used for comparison between Chinese and Dutch human milk, and the *p*-values for differences between them were plotted. (**A**) Significantly different proteins in Chinese and Dutch human milk over the course of lactation, based on difference in slope; (**B**) significantly different proteins in Chinese and Dutch human milk at week 1, based on intercept; and (**C**) no significant difference.

The differences in protein patterns between Chinese and Dutch human milk were examined by comparison of both the intercept (representing colostrum) and slope (representing the decline over lactation) of curves, fitted for the 166 common milk serum proteins. The *p*-values for these differences after using a two-tailed *t*-test are shown in Figure 5.

The levels of two serum proteins (elongation factor 2 and myristoylated alanine-rich c-kinase substrate) varied in the Chinese and Dutch human milk over the course of lactation, as shown by the significantly different slope (Figure 5, area A). Next to that, the levels of 35 serum proteins varied in intercept (Figure 5, area B), including several proteins from the top 15 (Table 1), as shown in green. The complete list of significantly different serum proteins in Chinese and Dutch human milk is shown in Table 2, grouped according to their biological function. 

The levels of the 166 common milk serum proteins in the Chinese and Dutch populations that increased or decreased over the course of lactation, can be found as supporting information (Table S1). The levels of 17 (10%) and 21 (12%) of the 166 common milk serum proteins changed over the course of lactation in Chinese and Dutch human milk, respectively. In addition, the 166 common serum proteins were compared between Chinese and Dutch human milk for each week separately (Table S2). This showed that 16 of 17 proteins that significantly differed in week 1 were also significantly differing in one or more of the other weeks.

## 4. Discussion

### 4.1. The Level and Type of Serum Proteins in Chinese Human Milk 

The total protein concentrations decrease significantly over a 20-week lactation period in each mother, although with individual variations (Figure 1). These milk serum protein concentrations match with those observed in earlier studies, ranging from 12 to 25 g/L [7,32,33,34], although other studies report lower values from 7 to 16 g/L over the course of lactation [3,24,35,36]. These differences may be explained by the BCA method [37,38], which generally overestimates the total protein in human milk by about 25–40% [37,38]. The serum protein levels in this study should thus be regarded as semi-quantitative, although this did not influence the comparisons reported here, as they are all based on the BCA method. Although the protein content seems high for milk serum, it should be taken into account that the samples with the highest protein content are actually those in early lactation. These samples are known to have higher protein and relatively lower casein contents [6], leading to higher milk serum protein contents. In addition, part of the casein remained in the sample after sample preparation and therefore also counted towards the BCA protein content.

As described previously [5], human milk becomes fully mature between 4 and 6 weeks postpartum, with the amounts of bioactive components decreasing relative to the nutrients. In early life, infants have an immature intestinal immune system, making them more vulnerable to infection by opportunistic pathogens [5]. The high levels of immune-related milk serum proteins in colostrum (Figure 3) may provide protection to the infant in this sensitive stage of development. 

It was also observed that a large variability exists in the milk serum protein composition in colostrum among Chinese mothers (Figure 2). The results in this study comprising milk from seven mothers shows that immune-active proteins, enzymes, and transport proteins are highly abundant in Chinese human milk (Figure 3), which can also be observed from the individual data of mothers (Figure 2). Earlier studies had already shown that immune-active proteins, enzymes, and transport proteins were present in the largest quantities over the course of lactation [7,9,11]. 

### 4.2. The 15 Most Abundant Milk Serum Proteins

The large quantities of immune-active proteins are especially driven by the abundance of lactoferrin, immunoglobulins, polymeric immunoglobulin receptor, clusterin, osteopontin and β_2_-microglobulin (Table 1), which may protect infants against pathogenic microorganisms, and confer passive immunity to the neonate until its own immune system has been developed [9,10,11]. As shown in Table 1, transport proteins, like free soluble caseins, serum albumin, and fatty acid binding protein were present in large quantities during lactation. Free soluble caseins could not be removed from the milk, unlike the micellar casein that can be pelleted by ultracentrifugation–a phenomenon that has also been reported by others [7,19,24]. Free soluble and micellar caseins belong to the most abundant proteins in human milk, and these proteins mainly supply infants with amino acids and minerals needed for their growth [23,24,25]. It can also be observed from Table 1 that enzymes are the largest group of proteins across lactation. The large quantities of enzymes in human milk can be explained by the presence of α-lactalbumin, which is known to be the most abundant milk serum protein (Table 1). This enzyme is required for the synthesis of lactose, the main macronutrient in milk [5,21]. It should be noticed that α-lactalbumin does not have enzymatic activity on its own. Besides α-lactalbumin, bile salt-activated lipase belongs to the 15 most important enzymes in Chinese and Dutch human milk during lactation (Table 1). Bile salt-activated lipase supports the digestion of fats in the immature infant digestive tract, and facilitates the absorption of cholesterol, vitamin A, and triacylglycerols [7]. The protease inhibitor α_1_-antichymotrypsin is also among the 15 most abundant human milk serum proteins, and, like other protease inhibitors and proteases, might play a key role in the digestion of human milk [12,13,14]. Overall, the 15 most abundant proteins identified in this study were in levels dominating the entire milk composition, covering more than 95% of both the Chinese and Dutch milk serum proteomes. 

### 4.3. Proteases and Protease Inhibitors

Proteases may play a key role in the digestion of human milk. Although trypsin was the most abundant protease in Chinese and Dutch human milk, many other proteases (e.g., cytosol aminopeptidase, elastase, kallikrein, plasmin, cathepsins) were found, albeit to a lesser extent (Supplementary Information, data file). As described by others, proteases might be present in human milk to hydrolyze proteins in the mammary gland to regulate casein micelle size [14,15]. Protein digestion in human milk by proteases target specific proteins (e.g., caseins, polymeric immunoglobulin receptor, osteopontin) that do not have an extensive tertiary structure and are thus more accessible to proteolytic cleavage [16,18]. These proteins were, in this study, part of the overall 15 most abundant proteins in Chinese and Dutch human milk during lactation (Table 1). In particular, the caseins are well digested [16,17,18], which indicates that proteases and bile salt-activated lipase in human milk aids overall in the digestion of two of its main macronutrients, fats and proteins [19]. 

Besides proteases, human milk also contains protease inhibitors. The ratio between protease inhibitors and proteases in colostrum is circa 10:1. The most abundant protease inhibitors were α_1_-antichymotrypsin, α_1_-antitrypsin, cystatin C, and phosphatidyletanolamine-binding protein (Supplementary Information, data file). As described by others, α_1_-antichymotrypsin binds to chymotrypsin and other chymotrypsin-like serine proteases in human milk, while α_1_-antitrypsin inhibits proteases, such as trypsin, elastase, plasmin, and thrombin, and irreversibly deactivates trypsin in vitro [12,13,14,15]. A correlation was found between protease inhibitors and immune-active proteins in Chinese and Dutch human milk (Figure 4). Previous literature focused specifically on the relation between serine protease inhibitors and immunoglobulins [7], which also in our data showed stronger correlations than for all protease inhibitors and all immune proteins (Figure S3). A correlation higher than 0.7 was also found in both Chinese and Dutch milk between proteases and protease inhibitors specifically (data not shown). A previous study presented an overview of the proteolytic system network in human milk [15], which consists of several proteases, protease inhibitors, and blood coagulation proteins, indicating that these protein groups share a common biochemical pathway; this may explain their correlations.

Where some of the major proteins are partially digested by milk proteases in human milk, most immune-active proteins are less sensitive to digestion by these proteases, due to their compact folded globular structure, that cannot be as easily digested [16]. For these immune-active proteins to have an immune-activating role in the small intestine, they must be protected against intestinal digestion, because they are sensitive to chymotrypsin and trypsin [17,18]. That might be the reason why protease inhibitors present in human milk seem to target intestinal enzymes, specifically blocking trypsin, chymotrypsin, and other proteases [17,18], especially through the relative abundant α_1_-antichymotrypsin and α_1_-antitrypsin. Overall, protease inhibitors may thus ensure that specific proteins stay intact in the infant’s digestive tract. This may also explain previous findings that several immune-active proteins (e.g., lactoferrin, lysozyme, immunoglobulins) and protease inhibitors (e.g., α_1_-antichymotrypsin, α_1_-antitrypsin) can be found intact in the stool of breastfed infants [17,18]. The intact proteins in the infant’s stool may also be related to the simultaneous decrease in the content of immune-active proteins and protease inhibitors over lactation. Protection is less necessary later in lactation due to the development of the infant’s immune system and digestive tract over time, while digestion becomes important for the release of nutrients later in lactation. 

### 4.4. Comparison of High- and Low- Abundance Serum Proteins in Chinese and Dutch Human Milk 

It appears that the milk serum proteomes of Chinese and Dutch mothers are similar (Figure 3 and Figure S2). The main purpose of this study was to evaluate the common serum proteins in Chinese and Dutch human milk over the course of lactation. Totals of 469 and 200 serum proteins were found in Chinese and Dutch human milk, respectively. Although a lower number of serum proteins was identified in Dutch human milk, there was still an overlap of 166 serum proteins with Chinese human milk, which represents more than 95% of the milk serum proteome in term of concentrations. The reason for the higher number of serum proteins found in Chinese human milk might be due to the larger sample size (48 versus 24 human milk samples), which generally leads to more identified proteins [28]. 

In total, 22% (37 out of 166) of the common serum proteins in human milk differed between Chinese and Dutch mothers either at week 1 or over the course of lactation. The levels of 35 of the 166 (circa 21%) common serum proteins varied between Chinese and Dutch mothers in week 1 (Figure 5, area B). This, together with the results presented in Table 2 and Table S2, indicates that the differences between Chinese and Dutch human milk serum proteins were mainly in their level throughout lactation, and not in their changes over lactation, as the levels of only 2 of the 166 (circa 1%) common serum proteins identified in this study (myristoylated alanine-rich c-kinase substrate and elongation factor 2) differed over the course of lactation (Figure 5, area A, showing difference in slope). Overall, the main differences in the milk serum proteomes between Chinese and Dutch human milk were observed in the level of individual proteins, and not in rate of changes over lactation.

## 5. Conclusions

The milk serum proteome of Chinese and Dutch mothers were similar in term of relative the abundance of different functional groups as well as the most abundant proteins. Some quantitative differences were found, especially in absolute levels and not in rates of change over lactation. Human milk contains enzymes that can assist the digestion of milk proteins and lipids in the immature infant’s digestive tract. Protease inhibitors, which are highly correlated to the immune-active proteins, are present in variable amounts in human milk; they could be relevant during digestion and might be involved in controlling protein breakdown in the infant’s intestinal tract. 

## Figures and Tables

**Table 1:** The 15 most abundant serum proteins categorized per function in both Chinese and Dutch human milk during lactation, with their corresponding BCA equivalent values (g/L) values at week 1.

Function	Protein Name	BCA Equivalent Values (g/L)	
		Chinese	Dutch
Enzyme	α-lactalbumin	6.98	8.73
	Bile salt-activated lipase	0.29	0.19
Immunity	Lactoferrin	3.74	2.10
	Ig α_1_-chain c-region	0.91	0.71
	Ig λ_2_-chain c-region	0.47	0.54
	Ig κ-chain c-region	0.39	0.90
	Polymeric immunoglobulin receptor	0.41	0.39
	Clusterin	0.23	0.17
	Osteopontin	0.17	0.19
	β_2_-microglobulin	0.16	0.16
Protease inhibitors	α_1_-antichymotrypsin	0.11	0.08
Transport	β-casein ^†^	1.17	3.91
	α_S1_-casein ^†^	1.33	1.34
	Serum albumin	0.93	1.06
	κ-casein ^†^	0.23	0.29
	Fatty acid-binding protein	0.07	0.13

**^†^** Micellar caseins were completely removed, while this was not the case for the free soluble part of the caseins.

**Table 2:** Significantly different serum proteins in Chinese and Dutch human milk, with *p*-values for week 1 (intercept) and over the course of lactation (slope).

Function *	Protein Name	*p*-Values of Serum Proteins (Chinese Versus Dutch)	
		Intercept	Slope
Cell	Actin	0.002 ^*^	0.540
	Calreticulin	0.000 ^*^	0.620
	Follistatin-related protein 1	0.003 ^*^	0.140
	MARCKS-like protein 1	0.004 ^*^	0.959
	Protein deglycase DJ-1	0.000 ^*^	0.051
	Peroxiredoxin 2	0.002 ^*^	0.233
Enzyme	4-trimethylaminobutyraldehyde dehydrogenase	0.000 ^*^	0.590
	**α-lactalbumin**	0.010 ^*^	**0.922**
	Fructose-bisphosphate aldolase A	0.000 ^*^	0.710
	Isocitrate dehydrogenase 1	0.000 ^*^	0.310
	L-lactate dehydrogenase A	0.000 ^*^	0.772
	Nucleoside diphosphate kinase A	0.000 ^*^	0.082
	Protein disulfide-isomerase	0.000 ^*^	0.685
	Transketolase	0.023 ^*^	0.707
	Triosephosphate isomerase	0.000 ^*^	0.912
	Tryptophan-tRNA ligase	0.000 ^*^	0.131
	UTP-glucose 1-phosphate uridylyltransferase	0.000 ^*^	0.630
Immunity	Complement C4B	0.000 ^*^	0.200
	**Ig α_1_-chain c-region**	0.000 ^*^	**0.210**
	Ig γ_3_ chain c-region	0.000 ^*^	0.190
	**Ig **κ**-chain c-region**	0.001 ^*^	**0.490**
	**Ig λ_2_-chain c-region**	0.045 ^*^	**0.640**
	Granulins	0.018 ^*^	0.800
	Lysozyme C	0.000 ^*^	0.937
	Monocyte differentiation antigen CD14	0.015 ^*^	0.770
Protease inhibitors	Inter-α-trypsin inhibitor heavy chain H2	0.000 ^*^	0.522
Protein synthesis	Elongation factor 2	0.547	0.050^*^
Signaling	14-3-3 protein β/α	0.000 ^*^	0.372
Transport	Apolipoprotein E	0.036 ^*^	0.500
	**β-casein ^†^**	0.000 ^*^	**0.590**
	**Fatty acid-binding protein**	0.000 ^*^	**0.790**
	Heat shock protein HSP 90-beta	0.040 ^*^	0.810
	**κ-casein ^†^**	0.038 ^*^	**0.950**
	Selenium-binding protein 1	0.006 ^*^	0.536
	**Serum albumin**	0.031 ^*^	**0.760**
	Transcobalamin 1	0.000 ^*^	0.509
Other	Myristoylated alanine-rich c-kinase substrate	0.001	0.028 ^*^

Bold type indicates the proteins also displayed in Table 1. **^†^** Micellar caseins were completely removed, while this was not the case for the free soluble part of the caseins. * Corresponding *p*-values (two-tailed *t*-test, α < 0.05)

## Supplementary Materials

The following are available online at https://www.mdpi.com/2072-6643/11/3/499/s1, Figure S1: Total BCA serum protein concentrations (g/L) in Dutch human milk per mother over a 24-week lactation period. Raw data from Dutch human milk were re-used [7]; Figure S2: BCA equivalent values (g/L) of serum proteins in human milk of 4 Dutch mothers categorized per biological function over a 24-week lactation period. Raw data from Dutch human milk were re-used [7]; Figure S3: Correlations between the functional groups consisting of protease inhibitors (including serine and non-serine protease inhibitors) and immune-active proteins (including immunoglobulins and non-immunoglobulins) in Chinese human milk, using BCA equivalent values (g/L) over a 20-week lactation period; Table S1: Significantly different serum proteins in Chinese and Dutch human milk over the course of lactation, based on the BCA equivalent values (g/L) over lactation (slope); Table S2: Serum proteins that were significantly different in at least one of the lactation weeks. Numbers are the *p*-value for the difference between the Chinese human milk serum proteins and Dutch human milk serum proteins. To facilitate direct comparison between Chinese and Dutch data within this research, the time points 12 and 20 weeks postpartum were compared to week 16 and 24, respectively; Supporting Information, data file: Serum proteins in human milk of Chinese mothers over a 20-week lactation period. The columns described in the next tab are the individual proteins, their functions and their iBAQ values averaged for all mothers at weeks 1, 2, 4, 8, 12, and 20 postpartum.

## References

[1] Kramer M., Kakuma R. (2004). The optimal duration of exclusive breastfeeding: A systematic review. Adv. Exp. Med. Biol..

[2] Liao Y., Alvarado R., Phinney B., Lönnerdal B. (2011). Proteomic characterization of human milk whey proteins during a twelve-month lactation period. J. Proteome Res..

[3] Martin C., Ling P., Blackburn G. (2016). Review of infant feeding: Key features of breastmilk and infant formula. Nutrients.

[4] Gartner L., Morton J., Lawrence R., Naylor A., O’Hare D., Schanler R., Eidelman A. (2005). Breastfeeding and the use of human milk. Pediatrics.

[5] Elwakiel M., Hageman J.A., Wang W., Szeto I.M., van Goudoever J.B., Hettinga K.A., Schols H.A. (2018). Human milk oligosaccharides in colostrum and mature milk of Chinese mothers: Lewis positive secretor subgroups. J. Agric. Food Chem..

[6] Kunz C., Lönnerdal B. (1992). Re-evaluation of the whey protein/casein ratio of human milk. Acta Paediatr..

[7] Zhang L., de Waard M., Verheijen H., Boeren S., Hageman J., van Hooijdonk T., Vervoort J., van Goudoever J., Hettinga K. (2016). Changes over lactation in breast milk serum proteins involved in the maturation of immune and digestive system of the infant. J. Proteomics.

[8] Zhang L., van Dijk A., Hettinga K. (2016). An interactomics overview of the human and bovine proteome over lactation. Proteome Sci..

[9] Hettinga K., Van Valenberg H., De Vries S., Boeren S., Van Hooijdonk T., Van Arendonk J., Vervoort J. (2011). The host defense proteome of human and bovine milk. PLoS ONE.

[10] Hettinga K., Reina F., Boeren S., Zhang L., Koppelman G. (2015). Difference in the breast milk proteome between allergic and non-allergic mothers. PLoS ONE.

[11] Beck K., Weber D., Phinney B., Smilowitz J., Hinde K., Lönnerdal B., Korf I., Lemay D. (2015). Comparative proteomics of human and macaque milk reveals species-specific nutrition during postnatal development. J. Proteome Res..

[12] Chowanadisai W., Lönnerdal B. (2002). α_1_-Antitrypsin and antichymotrypsin in human milk: Origin, concentrations, and stability. Am. J. Clin. Nutr..

[13] Lindberg T. (1982). Protease inhibitors in human milk. Pediatr. Res..

[14] Dallas D., Murray N., Gan J. (2015). Proteolytic systems in milk: Perspectives on the evolutionary function within the mammary gland and the infant. J. Mammary Gland Biol. Neoplasia..

[15] Kelly A., O’Flaherty P., Fox P. (2006). Indigenous proteolytic enzymes in milk: A brief overview of the present state of knowledge. Int. Dairy J..

[16] Dingess K., de Waard M., Boeren S., Vervoort J., Lambers T., van Goudoever J., Hettinga K. (2017). Human milk peptides differentiate between the preterm and term infant and across varying lactational stages. Food Funct..

[17] Dallas D., Guerrero A., Khaldi N., Borghese R., Bhandari A., Underwood M. (2014). A peptidomic analysis of human milk digestion in the infant stomach reveals protein-specific degradation patterns. J. Nutr..

[18] Su M., Broadhurst M., Liu C., Gathercole J., Cheng W., Qi X. (2017). Comparative analysis of human milk and infant formula derived peptides following in vitro digestion. Food Chem..

[19] Lönnerdal B. (2004). Human milk proteins: Key components for the biological activity of human milk. Adv. Exp. Med. Biol..

[20] Lönnerdal B. (2010). Bioactive proteins in human milk: Mechanisms of action. J. Pediatr..

[21] Newburg D. (2001). Bioactive components of human milk: Evolution, efficiency and protection. Adv. Exp. Med. Biol..

[22] Spik G., Brunet B., Mazurier-Dehaine C., Fontaine G., Montreuil J. (1982). Characterization and properties of the human and bovine lactotransferrins extracted from the faeces of newborn infants. Acta Pædiatr. Scand..

[23] Davidson L., Lönnerdal B. (1987). Persistence of human milk proteins in the breastfed infant. Acta Pædiatr..

[24] Lönnerdal B. (2003). Nutritional and physiologic significance of human milk proteins. Am. J. Clin. Nutr..

[25] Jakaitis B., Denning P. (2014). Human breast milk and the gastrointestinal innate immune system. Clin. Perinatol..

[26] Jensen H., Poulsen N., Moller H., Stensballe A., Larsen L. (2012). Comparative proteomic analysis of casein and whey as prepared by chymosin-induced separation, isoelectric precipitation or ultracentrifugation. J. Dairy Res..

[27] Lu J., Boeren S., de Vries S., van Valenberg H., Vervoort J. (2011). Filter-aided sample preparation with dimethyl labeling to identify and quantify milk fat globule membrane proteins. J. Proteomics.

[28] Cox J., Mann M. (2008). MaxQuant enables high peptide identification rates, individualized ppb-range mass accuracies and proteome-wide protein quantification. Nature.

[29] Schwanhausser B., Busse D., Li N., Dittmar G., Schuchhardt J., Wolf J., Chen W., Selbach M. (2011). Global quantification of mammalian gene expression control. Nature.

[30] Huang D., Sherman B., Lempicki R. (2009). Systematic and integrative analysis of large gene lists using DAVID Bioinformatics Resources. Nat. Protoc..

[31] Tyanova S., Temu T., Sinitcyn P., Carlson A., Hein M., Geiger T., Mann M., Cox J. (2016). The Perseus computational platform for comprehensive analysis of proteomics data. Nat. Methods.

[32] Breckwoldt J., Neulen J., Keck C., Greger R., Windhorst U. (1996). Lactation. From cellular mechanisms to integration. Comprehensive Human Physiology.

[33] Monaco M., Donavan S., Walker W., Watkins J., Duggan C. (2008). Human milk: Nutritional properties. Nutrition in Pediatrics: Basic Science and Clinical Applications.

[34] Trend S., Strunk T., Hibbert J., Kok C., Zhang G., Doherty D., Richmond P., Burgner D., Simmer K., Davidson D. (2015). Antimicrobial protein and peptide concentrations and activity in human breast milk consumed by preterm infants at risk of late-onset neonatal sepsis. PLoS ONE.

[35] Liao Y., Weber D., Xu W., Durbin-Johnson B., Phinney B., Lönnerdal B. (2017). Absolute quantification of human milk caseins and the whey/casein ratio during the first year of lactation. J. Proteome Res..

[36] Lönnerdal B., Erdmann P., Thakkar S., Sauser J., Destaillats F. (2017). Longitudinal evolution of true protein, amino acids and bioactive proteins in breast milk: A developmental perspective. J. Nutr. Biochem..

[37] Wu X., Jackson R., Khan S., Ahuja J., Pehrsson P. (2018). Human milk nutrient composition in the United States: Current knowledge, challenges, and research needs. Curr. Dev. Nutr..

[38] Perrin M., Fogleman A., Newburg D., Allen J. (2017). A longitudinal study of human milk composition in the second year postpartum: Implications for human milk. Matern. Child Nutr..

