# Supplementary material for: Variability of Serum Proteins in Chinese and Dutch Human Milk during Lactation
Source: Nutrients. 2019 Feb 27;11(3):499. doi: 10.3390/nu11030499 (PMC6471199; doi:10.3390/nu11030499)
Supplement: Supplementary file 1 [file nutrients-11-00499-s001.zip › supplementary/supplementary.docx]

**Table S1.** Significantly different serum proteins in Chinese and Dutch human milk over lactation, based on the BCA equivalent values (g/L) over lactation (slope).

|  | **Function** | **Protein Name** | **ρ-value slope** |
| --- | --- | --- | --- |
| **Chinese** | Cell | Actin $(\downarrow)$ | 0.040 |
|  |  | Platelet glycoprotein 4 $(\downarrow)$ | 0.040 |
|  | Enzyme | Legumain $(\downarrow)$ | 0.047 |
|  |  | Lipoprotein lipase $(\downarrow)$ | 0.049 |
|  |  | Prosaposin $(\downarrow)$ | 0.042 |
|  |  | Triosephosphate isomerase $(\downarrow)$ | 0.045 |
|  | Immunity | Chitinase 3-like protein 1 $(\downarrow)$ | 0.042 |
|  |  | Ig heavy chain V-III-region TRO $(\downarrow)$ | 0.049 |
|  |  | Ig γ_3_-chain c-region $\boldsymbol{(}\boldsymbol{\downarrow)}$ | 0.050 |
|  |  | **Ig κ-chain c-region** $\boldsymbol{(}\boldsymbol{\downarrow)}$ | **0.046** |
|  |  | Lactadherin $\boldsymbol{(}\boldsymbol{\downarrow)}$ | 0.035 |
|  |  | Lactoferrin $(\downarrow)$ | 0.034 |
|  |  | Mucin 1 $\boldsymbol{(}\boldsymbol{\downarrow)}$ | 0.031 |
|  |  | Xanthine dehydrogenase $\boldsymbol{(}\boldsymbol{\downarrow)}$ | 0.044 |
|  | Protease inhibitor | Plasma protease C_1_-inhibitor $\boldsymbol{(}\boldsymbol{\downarrow)}$ | 0.043 |
|  | Signaling | G-protein coupled receptor family C group 5B $\boldsymbol{(}\boldsymbol{\downarrow)}$ | 0.048 |
|  | Other | Fibrinogen gamma chain $\boldsymbol{(}\boldsymbol{\downarrow)}$ | 0.045 |
| **Dutch*** | Cell | Galectin 3-binding protein $(\downarrow)$ | 0.013 |
|  |  | Leucine-rich α_2_-glycoprotein $(\downarrow)$ | 0.039 |
|  |  | Nucleobindin 2 $(\downarrow)$ | 0.003 |
|  |  | Tenascin $(\downarrow)$ | 0.018 |
|  | Enzyme | **Bile salt-activated lipase** $\boldsymbol{(}\boldsymbol{\downarrow)}$ | **0.010** |
|  |  | L-lactate dehydrogenase $\boldsymbol{(}\boldsymbol{\downarrow)}$ | 0.000 |
|  |  | Sulfhyldryl oxidase 1 $\boldsymbol{(}\boldsymbol{\downarrow)}$ | 0.031 |
|  |  | UTP-glucose 1 phosphate uridylyltransferase $\boldsymbol{(}\boldsymbol{\downarrow)}$ | 0.012 |
|  | Immunity | Haptoglobin $\boldsymbol{(}\uparrow)$ | 0.031 |
|  |  | Lysozyme C $\boldsymbol{(}\boldsymbol{\downarrow)}$ | 0.034 |
|  |  | Zinc α_2_-glycoprotein $\boldsymbol{(}\uparrow)$ | 0.002 |
|  | Protease inhibitors | α_1_-antitrypsin $\boldsymbol{(}\boldsymbol{\downarrow)}$ | 0.046 |
|  |  | Phosphatidylethanolamine binding protein 1 $(\downarrow)$ | 0.015 |
|  | Transport | **α_S_1-casein** $\boldsymbol{(}\boldsymbol{\downarrow)}$**^†^** | **0.006** |
|  |  | **Fatty acid-binding protein** $\boldsymbol{(}\uparrow)$ | **0.012** |
|  |  | **κ-casein** $\boldsymbol{(}\uparrow)$**^†^** | **0.010** |
|  |  | Selenium binding protein 1 $\boldsymbol{(}\boldsymbol{\downarrow)}$ | 0.025 |
|  |  | Serotransferrin $\boldsymbol{(}\boldsymbol{\downarrow)}$ | 0.018 |
|  |  | **Serum albumin** $\boldsymbol{(}\uparrow)$ | **0.001** |
|  | Other | Chordin-like protein 2 $(\downarrow)$ | 0.034 |
|  |  | Gelsolin $(\downarrow)$ | 0.011 |

**Bold:** Indicate the proteins displayed in Table 1. **^†^** Micellar caseins were completely removed, while this was not the case for the free soluble part of the caseins. Arrows are indicative for an increase or decrease over lactation for each protein. * Significantly different serum proteins in Dutch human milk over lactation were already previously reported [7].

Table S2: Serum proteins that were significantly different in at least one of the lactation weeks. Numbers are the p-value for the difference between the Chinese human milk serum proteins and Dutch human milk serum proteins. To facilitate direct comparison between Chinese and Dutch data within this research, the time points wk 12 and 20 postpartum were compared to wk 16 and 24, respectively.

| Protein names | Week 1 | Week 2 | Week 4 | Week 8 | Week 12/16 | Week 20/24 |
| --- | --- | --- | --- | --- | --- | --- |
| Ig γ_2_-chain c-region | **<0.0001** | **<0.0001** | **<0.0001** | **<0.0001** | **<0.0001** | ***0.0000*** |
| Ig μ-chain c-region | **<0.0001** | ***0.0113*** | ***0.0254*** | ***0.0032*** | ***0.0007*** | ***0.0457*** |
| Complement C4A | ***0.0001*** | ***0.0029*** | ***0.0298*** | ***0.0064*** | **<0.0001** | ***0.0031*** |
| Galectin-3-binding protein | ***0.0001*** | 0.7715 | 0.4929 | 0.3741 | 0.4760 | 0.0787 |
| **Ig α_1_-chain c-region** | ***0.0004*** | ***0.0002*** | **<0.0001** | **<0.0001** | ***0.0001*** | ***0.0001*** |
| Ig γ_3_-chain c-region | ***0.0010*** | ***0.0025*** | ***0.0011*** | ***0.0003*** | ***0.0009*** | ***0.0021*** |
| **Ig κ-chain c-region** | ***0.0016*** | 0.0764 | 0.5174 | ***0.0236*** | 0.2549 | 0.1576 |
| Mucin1 | ***0.0017*** | ***0.0328*** | 0.3881 | 0.6801 | 0.2955 | ***0.0190*** |
| Protein S100-A9 | ***0.0026*** | 0.1452 | 0.5719 | 0.0526 | ***0.0137*** | 0.1594 |
| Chordin-like protein 2 | ***0.0031*** | ***0.0035*** | 0.6800 | 0.8939 | 0.1924 | 0.5632 |
| Complement C4B | ***0.0041*** | ***0.0004*** | ***0.0100*** | ***0.0010*** | **<0.0001** | ***0.0005*** |
| Sclerostin domain-containing protein 1 | ***0.0048*** | ***0.0430*** | ***0.0183*** | 0.1233 | ***0.0204*** | 0.1964 |
| Apolipoprotein E | ***0.0050*** | ***0.0012*** | ***0.0038*** | ***0.0119*** | ***0.0091*** | 0.0550 |
| Transcobalamin-1 | ***0.0084*** | 0.1100 | 0.5721 | 0.1655 | 0.4203 | ***0.0004*** |
| Ezrin | ***0.0169*** | ***0.0100*** | ***0.0230*** | 0.0774 | ***0.0032*** | ***0.0229*** |
| Myristoylated alanine-rich C-kinase substrate | ***0.0196*** | 0.7578 | 0.1413 | 0.5531 | ***0.0030*** | ***0.0306*** |
| Apolipoprotein B-100 | ***0.0398*** | ***0.0147*** | ***0.0000*** | ***0.0489*** | 0.1484 | 0.1247 |
| **β-casein** | 0.0533 | 0.8070 | 0.7004 | ***0.0015*** | 0.8484 | ***0.0491*** |
| Ig γ_1_-chain c-region | 0.0589 | ***0.0114*** | ***0.0430*** | 0.4278 | ***0.0022*** | 0.6679 |
| Protein disulfide-isomerase | 0.1661 | ***0.0074*** | 0.1549 | 0.0599 | ***0.0121*** | ***0.0055*** |
| Selenium-binding protein 1 | 0.2163 | 0.1200 | 0.0577 | ***0.0235*** | ***0.0024*** | ***0.0011*** |
| 45 kDa calcium-binding protein | 0.3497 | 0.0942 | 0.2351 | ***0.0084*** | ***0.0047*** | 0.2351 |
| Ribonuclease T2 | 0.3545 | ***0.0076*** | ***0.0094*** | 0.9075 | ***0.0028*** | NA |
| Beta-2-glycoprotein 1 | 0.5483 | ***0.0002*** | 0.3685 | ***0.0230*** | 0.0545 | 0.6026 |
| Legumain | 0.6446 | 0.9789 | 0.6999 | 0.4638 | 0.8119 | ***0.0025*** |
| Complement C3 beta chain | 0.6921 | 0.1056 | 0.0542 | ***0.0422*** | ***0.0000*** | ***0.0197*** |
| Protein S100-A11 | 0.7731 | 0.8635 | ***0.0007*** | 0.8139 | 0.4929 | 0.0940 |
| Apolipoprotein D | 0.7739 | 0.0991 | 0.0657 | ***0.0006*** | 0.5928 | 0.3749 |
| Gamma-glutamyltranspeptidase 1 | 0.8008 | 0.2640 | ***0.0301*** | 0.2631 | 0.1578 | ***0.0024*** |
| Heat shock cognate 71 kDa protein | 0.9118 | 0.1782 | ***0.0241*** | 0.0977 | ***0.0002*** | ***0.0380*** |
| Triosephosphate isomerase | 0.9866 | ***0.0144*** | 0.0912 | 0.1336 | ***0.0028*** | ***0.0172*** |

**Figure S1.**


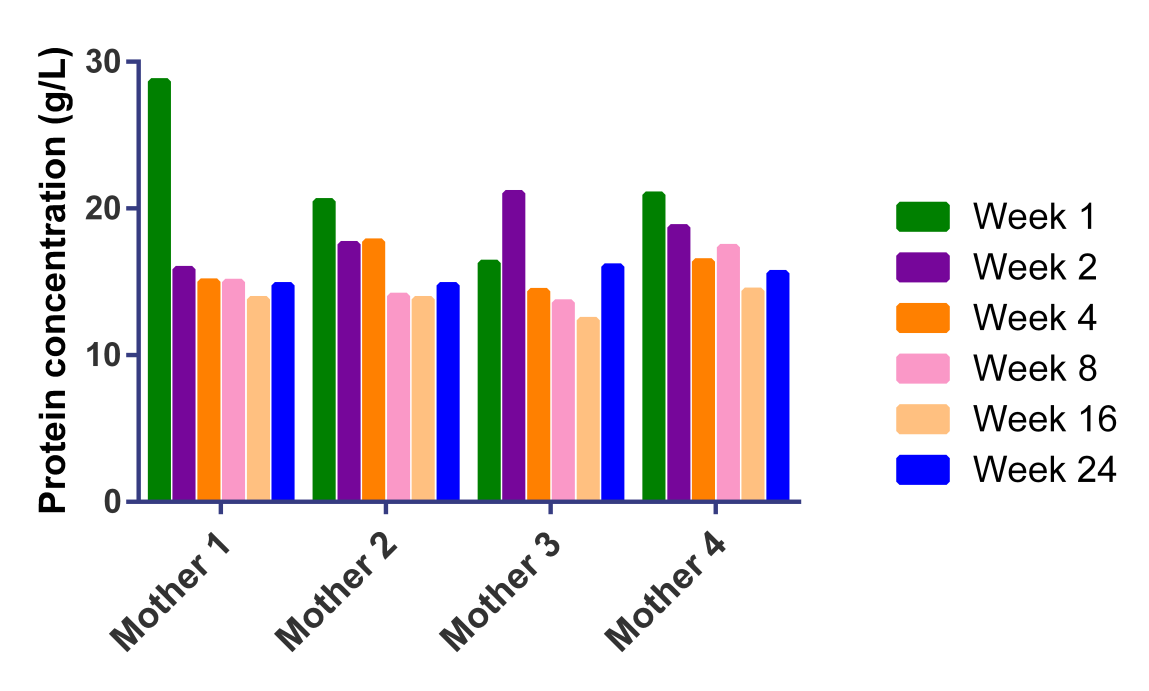


**Figure S1.** Total BCA serum protein concentrations (g/L) in Dutch human milk per mother over a 24-wk lactation period. Raw data from Dutch human milk was re-used [7].

**Figure S2.**

**
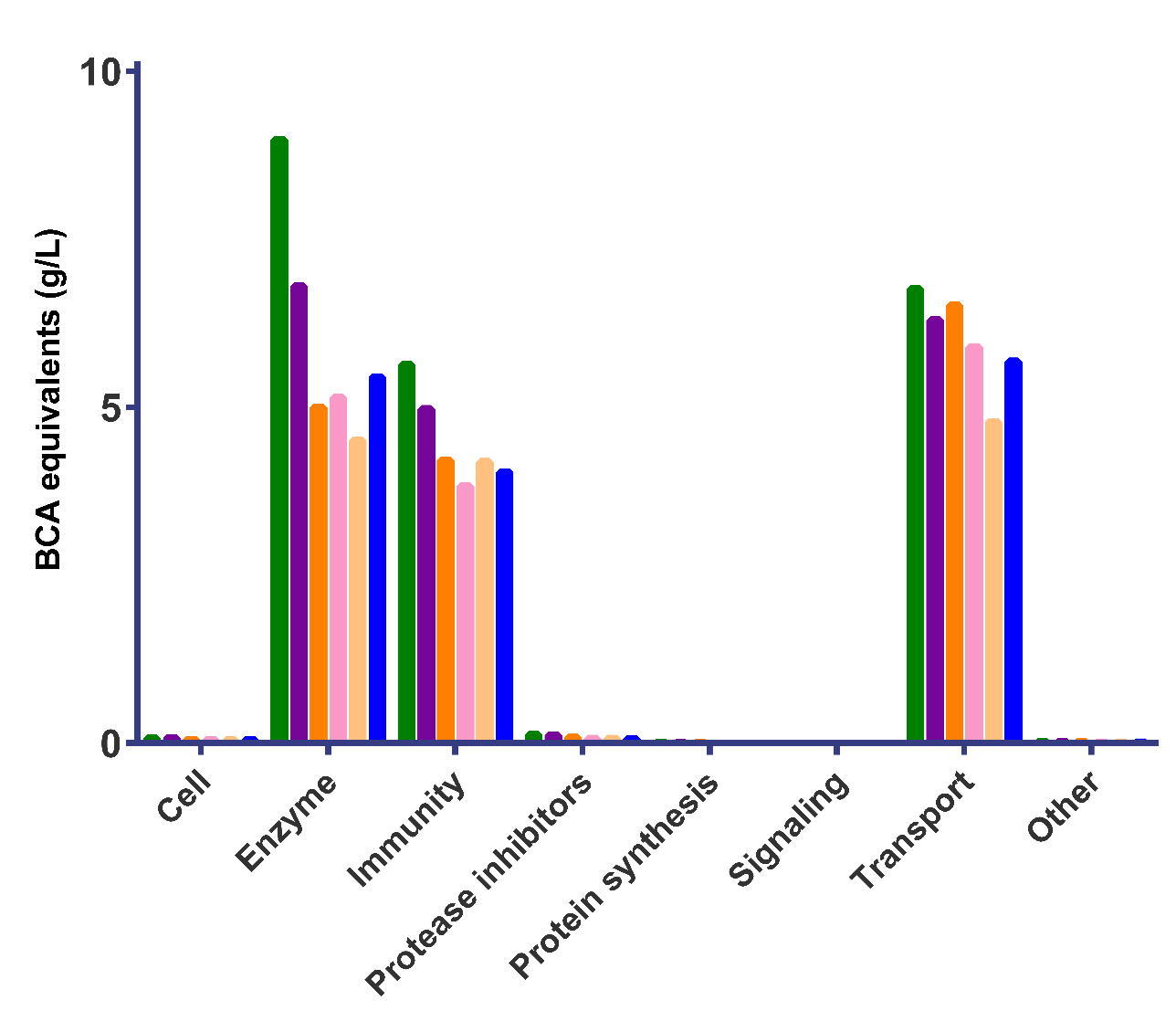
**

**Figure S2.** BCA equivalent values (g/L) of serum proteins in human milk of 4 Dutch mothers categorized per biological function over a 24-wk lactation period. Raw data from Dutch human milk was re-used [7].

**Figure S3.** Correlations between the functional groups consisting of protease inhibitors (including serine and non-serine protease inhibitors) and immune-active proteins (including immunoglobulins and non-immunoglobulins) in Chinese human milk, using BCA equivalent values (g/L) over a 20-wk lactation period.


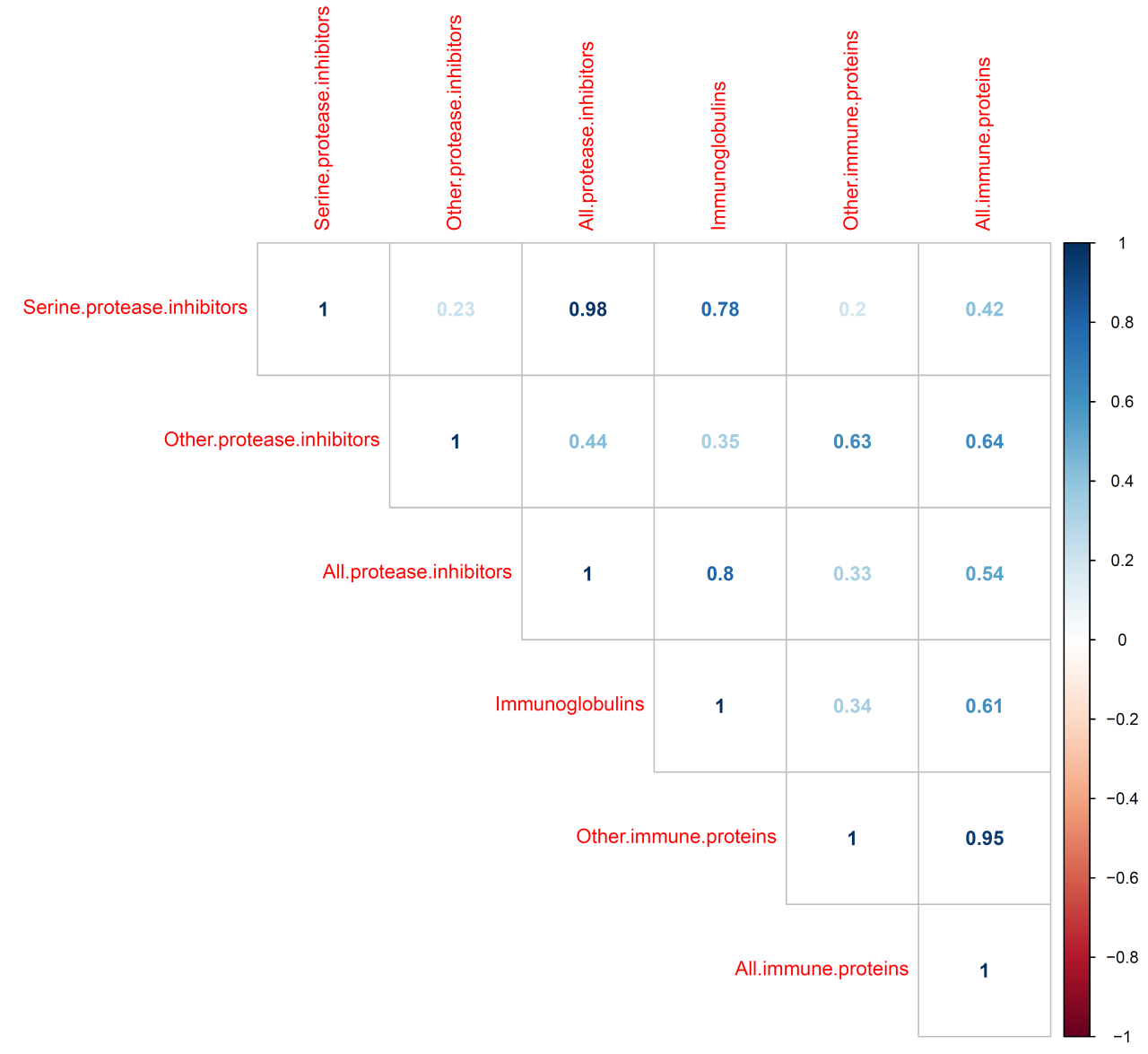


**Data file.**

Raw data: Serum proteins in human milk of Chinese mothers over a 20-wk lactation period.
